# Supplementary material for: Supervised training of spiking neural networks for robust deployment on mixed-signal neuromorphic processors
Source: Sci Rep. 2021 Dec 3;11:23376. doi: 10.1038/s41598-021-02779-x (PMC8642544; doi:10.1038/s41598-021-02779-x)
Supplement: Supplementary file 1 — Supplementary Information. [file 41598_2021_2779_MOESM1_ESM.pdf]

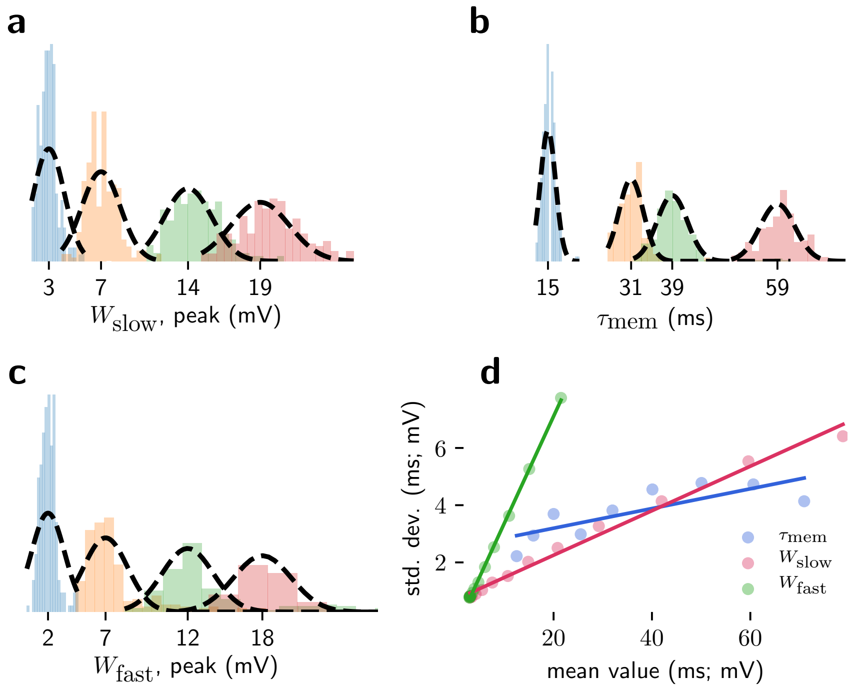

Fig. S1. **Neuronal and synaptic parameter mismatch on mixed-signal Neuromorphic devices follow a mean-to-variance linear scaling rule.** Measurements of actual weights of two types of silicon synapses with slow (**a**) and fast dynamics (**c**), as well as neuron time constants (**b**), show a consistent linear relationship between nominal set value and the distribution of actual mismatch parameter values. In all cases, the variance of measured parameters scaled with the nominal set value (**d**) (linear regression $r = 0.79$ τ_{mem}; $r = 0.994$ W_{slow}; $r = 0.9996$ W_{fast}).

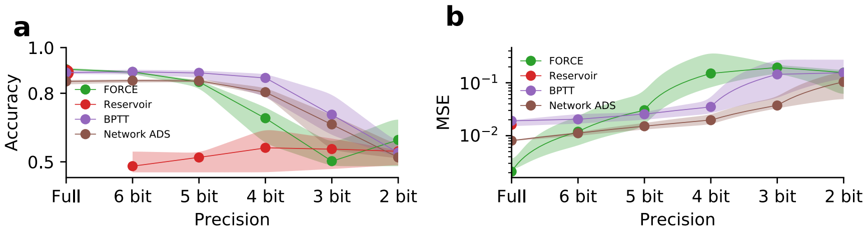

Fig. S2. **Our method is more robust to quantisation noise than other standard training approaches.** Median and IQR for accuracy (**a**) and MSE (**b**) for four network architectures. The weights of 10 networks for each architecture were quantised to the bit-depths indicated, then evaluated on 1000 test samples each. Reservoir performance degraded completely for all quantisation bit-depths (very high MSE) so is not indicated in **b**.

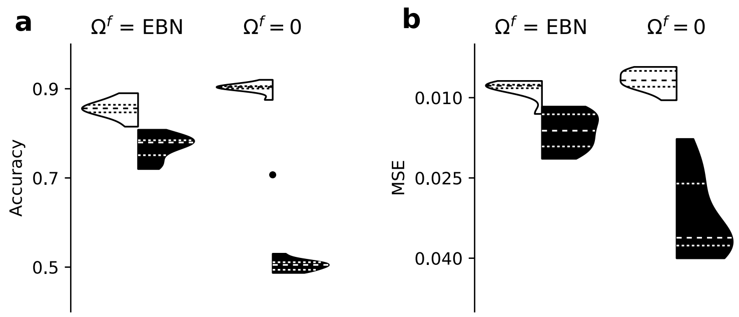

Fig. S3. **Fast balanced feedback $\Omega^{\mathbf{f}}$ provides robustness to neuron silencing.** The effect of silencing 40% of neurons in spiking ADS networks is shown on accuracy (**a**) and on output error MSE (**b**). White: Network performance without silencing. Black: Network performance when silencing 40% of spiking neurons. Fast balanced recurrent feedback ($\Omega^{\mathbf{f}}$ = EBN) provided robustness to neuron silencing (small drop in performance). Networks without fast balanced recurrent feedback ($\Omega^{\mathbf{f}} = 0$) exhibited more severe performance degradation under neuron silencing (large drop in performance).

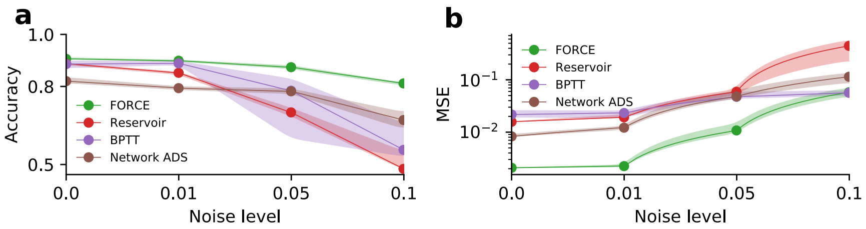

Fig. S4. **Effect of membrane potential noise on network responses.** Median and IQR for accuracy (**a**) and MSE (**b**) for four network architectures. Noise was added to 10 instances of each network architecture, as Normally-distributed noise with std. dev. σ scaled to the range between V_{thresh} and V_{reset} for each neuron (see Methods).

TABLE S1

POWER ESTIMATIONS FOR A NON-SPIKING RNN AND THE CORRESPONDING SNN IMPLEMENTED ON DYNAP-SE1.

ANN neurons $\hat{N}$	STM32L552xx[47] @16 MHz (mW)	STM32L552xx[47] @80 MHz (mW)	EIE[48] @65 nm (mW)	DYNAP-SE1 @65 nm (mW)
32	0.4	0.45	**0.027**	0.032
64	1.13	1.31	0.11	**0.038**
128	N/A†	4.8	0.41	**0.057**
256	N/A†	11.4	1.6	**0.13**
512	N/A†	N/A†	6.5	**0.43**

†For these parameter combinations, the RNN required more computation than possible on the MCU for real-time operation.

Supplementary Methods

Learning in adaptive non-linear control theory

Let us assume an arbitrary dynamical system of the form

$$\dot{\mathbf{x}}(t) = f(\mathbf{x}(t)) + \mathbf{c}(t) \tag{1}$$

where $\mathbf{x}(t)$ is a vector of state variables $x_j(t)$, $f(.)$ is a non-linear function (e.g. $\tanh(.)$), and $\mathbf{c}(t)$ is a time-dependent input of the same dimensionality of $\mathbf{x}(t)$. Furthermore, let us assume a "student" dynamical system of the form

$$\dot{\hat{\mathbf{x}}}(t) = -\lambda\hat{\mathbf{x}}(t) + \mathbf{W}^T\Psi(\hat{\mathbf{x}}(t)) + \mathbf{c}(t) + k \cdot \mathbf{e}(t) \tag{2}$$

where $\hat{\mathbf{x}}(t)$ is a vector of state variables $\hat{x}_j(t)$, λ is a leak term, and $\mathbf{c}(t)$ is the same time-dependent input as in Equation 1. Over time, the signed error $\mathbf{e}(t) = \mathbf{x}(t) - \hat{\mathbf{x}}(t)$ between the teacher dynamics (Eq. 1) and the student dynamics (Eq. 2) is computed and fed into the student dynamics, causing the student state variables $\hat{\mathbf{x}}(t)$ to closely follow the target variables $\mathbf{x}(t)$.

This close tracking enables us to update the weights $\mathbf{W}$ using Eq. 3, so that over the course of learning, the factor k can be reduced to zero and the network follows the teacher dynamics autonomously, using only a weighted sum of basis functions, given by $\Psi(\hat{\mathbf{x}}(t)) = \phi(\mathbf{M}\hat{\mathbf{x}}(t) + \theta)$, for some non-linear function ϕ, and some random $\mathbf{M}$ and θ.

The learning rule used to adapt the weights $\mathbf{W}$ is given by

$$\dot{\mathbf{W}} = \eta\Psi(\hat{\mathbf{x}}(t))\mathbf{e}(t)^T \tag{3}$$

and can be shown to let the weights $\mathbf{W}$ converge towards the optimal weights, denoted $\mathbf{W}^{\text{true}}$, assuming that the input $\mathbf{c}(t)$ does not lie on a low-dimensional manifold and that the student system has enough high-dimensional basis functions. For more information and a proof of the above statement, see ref. 41

It should be noted that the relation between $x(t)$ and $c(t)$ should be well-defined by an autonomous (non-)linear dynamical system of the form $\dot{\mathbf{x}}(t) = f(\mathbf{x}(t)) + \mathbf{c}(t)$ and that one cannot simply assume a "black box" dynamical system implementing *any* relation of the form $\dot{\mathbf{x}}(t) = \mathcal{B}(\mathbf{x}(t), \mathbf{x}(t))$. Concretely, in the light of classification, one cannot simply

assume there exists an autonomous dynamical system relating the input $\mathbf{c}(t)$ to some target response variable, and that this relation can be learned by the learning rule described above. This observation is important, as it makes it harder to build a classifier given the tools described above. In the next section, we will review how a network of spiking neurons can implement the above learning rule in order to learn the dynamics of a teacher dynamical system.

Learning arbitrary dynamical systems in Efficient Balanced Networks (EBNs)

In this section, we will briefly recapitulate how an EBN of spiking neurons can learn to implement any non-linear dynamical system of the form $\dot{\mathbf{x}}(t) = f(\mathbf{x}) + \mathbf{c}(t)$. We will assume that, given a network of N neurons, one can use a decoder $\mathbf{D}$ to reconstruct the target variable from the filtered spike trains of the population using $\hat{\mathbf{x}}(t) = \mathbf{D}\mathbf{r}(t)$, so that $\mathbf{x}(t) \approx \hat{\mathbf{x}}(t)$.

Derived from the fact that in an EBN a neuron only fires a spike if it contributes to reducing the loss L,[37,38] given by

$$L = \frac{1}{T}\sum_{t=0}^{T} \|x(t) - \hat{x}(t)\|_2^2 + \mu\|r(t)\|_2^2 + \nu\|r(t)\|_1$$

with smoothed firing rates $r(t)$ and loss regularisation terms μ and ν, the membrane potentials in the network are then given by

$$V(t) = \mathbf{D}^T\mathbf{x}(t) - \mathbf{D}^T\mathbf{D}\mathbf{r}(t) - \mu\mathbf{r}(t) \tag{4}$$

Following ref. 41, we differentiate Eq. 4 and substitute the smoothed firing rates $\dot{\mathbf{r}}(t) = -\lambda\mathbf{r}(t) + \mathbf{o}(t)$; the teacher dynamics $\dot{\mathbf{x}}(t) = f(\mathbf{x}(t)) + \mathbf{c}(t)$; and the decoded dynamics of the student $\hat{\mathbf{x}}(t) = \mathbf{D}\mathbf{r}(t)$, to obtain

$$\begin{aligned}\dot{V}(t) &= -\lambda V(t) + \mathbf{D}^T(f(\mathbf{x}(t) + \mathbf{c}(t))) - \mathbf{D}^T\mathbf{D}(-\lambda\mathbf{r}(t) + \mathbf{o}(t)) - \mu(-\lambda\mathbf{r}(t) + \mathbf{o}(t)) \\ &= -\lambda V(t) + \mathbf{D}^T\mathbf{c}(t) - (\mathbf{D}^T\mathbf{D} + \mu\mathbf{I})\mathbf{o}(t) + \mathbf{D}^T(\lambda\mathbf{x}(t) + f(\mathbf{x}(t)))\end{aligned}$$

with decay rates λ; population spike trains $\mathbf{o} = V > V_{\text{thresh}}$; and identity matrix $\mathbf{I}$.

Under the non-linear control learning theoretical result of ref. 41, the term $\lambda\mathbf{x}(t) + f(\mathbf{x}(t))$ is approximated by a weighted set of basis functions over slow recurrent feedback weights Ω^{s}, given by $\Omega^{\text{s}}\Psi(\mathbf{r}(t))$, where $\Psi(\mathbf{r}(t)) =$

$\phi(\mathbf{Mr}(t)+\theta)$, inspired by complex multi-compartmental dendritic dynamics. In our networks we omitted the term $\Psi(\mathbf{r}(t))$ and simply replaced it with the population spike train $\mathbf{o}(t)$.

By feeding the error term $\mathbf{e}(t) = \mathbf{x}(t) - \hat{\mathbf{x}}(t)$ back into the network using the encoding/decoding weights $\mathbf{D}^T$, we obtain the final network dynamics

$$\dot{V}(t) = -\lambda V(t) + \mathbf{Fc}(t) - \Omega^{\mathbf{f}}\mathbf{o}(t) + \Omega^{\mathbf{s}}\mathbf{o}(t) + k\mathbf{D}^T \mathbf{e}(t)$$

where the encoding weights $\mathbf{F}$ are given by $\mathbf{D}^T$ and the optimal fast recurrent weights by $\Omega^{\mathbf{f}} = -(\mathbf{D}^T\mathbf{D} + \mu\mathbf{I})$.[38]

Similar to the case in control-theory, the learning rule for the slow recurrent weights is given by

$$\dot{\boldsymbol{\Omega}}^{\mathbf{s}} = \eta\Psi(\mathbf{r}(t))(\mathbf{D}^T\mathbf{e}(t))^T$$

where we simply replaced $\Psi(\mathbf{r}(t))$ with $\mathbf{r}(t)$.

To speed up the simulations, one could assume that the accumulated updates to $\Omega^{\mathbf{s}}$, given by $\sum_{t=0}^{T}\eta\mathbf{r}(t)(\mathbf{D}^T\mathbf{e}(t))^T$ are approximately the same as accumulating the rates and errors into large arrays of size $N \times T$ and performing the update in a batched fashion, after a whole signal was evaluated rather than after every time-step, using

$$\dot{\Omega}^{\mathbf{s}} = \eta\mathbf{r}(\mathbf{D}^T\mathbf{e})^T$$

We verified experimentally that this method is indeed faster, but yields less optimal results.

Spiking ADS network connectivity

The decoder weights, denoted $\mathbf{D}$, are initialised using a standard normal distribution $\mathcal{N}(0, 1/N_{\mathbf{c}})$, where $N_{\mathbf{c}}$ is the dimensionality of the input signal. The encoding weights $\mathbf{F}$ are given by simply transposing the decoding weights such that $\mathbf{F} = \mathbf{D}^T$.

Unless stated otherwise, the slow recurrent connections were initialized using a zero matrix: $\Omega^{\mathbf{s}} = \mathbf{0}$.

Following the optimal network connectivity of EBNs,[38] we initialized $\Omega^{\mathbf{f}_*}$ with $\mathbf{D^T D} + \mu\lambda_d^2 \mathbf{I}$, which was then transformed to $\Omega^{\mathbf{f}}$ using

$$\Omega^{\mathbf{f}} = \frac{\xi a}{\tau_{\text{fast}}} \Omega^{\mathbf{f}_*} / V^*_{\text{thresh}}$$

where

$$V^*_{\text{thresh,n}} = \frac{\nu\lambda_d + \mu\lambda_d^2 + ||D_n||_2^2}{2}$$

with $\mu = 0.0005$, $\nu = 0.0001$, $\lambda_d = 20$, $\xi = 10$, and $a = 0.5$.

We applied the transformation described in the section "Scaling and physical units" in ref.[38] to transform our network to have $V_{\text{reset}} = 0$ and $V_{\text{thresh}} = 1.0$.

Training an EBN-based classifier

The coding properties of EBNs make them attractive for many applications, especially in the neuromorphic community. However, so far it has only been shown how to implement linear and non-linear dynamical systems of a specific form using EBNs. In this section we provide a method to train an EBN to perform classification tasks of varying complexity.

Let us define our time-varying, real-valued input as $\mathbf{c}(t)$, where $\mathbf{c} \in \mathcal{R}^{N_c \times 1}$ and $t \in \{0...T\}$. The goal of training a classifier is to find a mapping $f(\mathbf{c}, \Theta) \rightarrow \mathbf{y}$ that maps any input $\mathbf{c}$ to the desired target variable $\mathbf{y}$, where $\mathbf{y}$ is a variable over time indicating the target and Θ is the set of system parameters. For simplicity, we will consider the case of binary classification. We however note that our method can be easily extended to a multi-class classification task.

Considering the ability of the learning rule presented in section , one might be be inclined to assume a "black box" dynamical system $\mathcal{B}$ of the form $\dot{\mathbf{y}}(t) = f(\mathbf{y}(t)) + \mathbf{c}(t)$ that, given input $\mathbf{c}$, autonomously produces the desired target $\mathbf{y}$. Two problems come with this approach:

1) The dynamical system is not well-defined, as it is no-longer explicitly defined by a teacher dynamical system. Furthermore, the system is autonomous, as the function $f(\cdot)$ does not depend on the input, but

only on past values of the target variable, making it impossible for the system to find a complex relationship between input and target.
2) This approach assumes that the input and target variable have the same number of dimensions, which is almost never the case.

How can we use the above learning rule to train an EBN to perform arbitrary classification tasks at low metabolic cost, high robustness and good classification performance? To answer this question, we consider a simple RNN comprising $\hat{N}$ units, following

$$\tau_j \dot{x}_j(t) = -x_j(t) + \hat{\mathbf{F}}c(t)_j + \hat{\mathbf{\Omega}} f(x(t))_j + b_j + \epsilon_j \tag{5}$$

where τ_j is the time constant of the j-th unit; $\hat{\mathbf{F}}$ are feed-forward encoding weights of shape $\hat{N} \times N_c$; $c(t)$ is the N_c dimensional input at time t; $f(.)$ is a non-linear function (e.g. tanh(.)); $\hat{\mathbf{\Omega}}$ are recurrent weights of shape $\hat{N} \times \hat{N}$; and b_j and ϵ_j are bias and noise terms, respectively.

We observe that an RNN can be rewritten in the general form

$$\begin{aligned}
\dot{\mathbf{x}}(t) &= \tilde{f}(\mathbf{x}) + \tilde{\mathbf{c}}(t), \text{with} \\
\tilde{f}(\mathbf{x}) &= 1/\tau(-\mathbf{x}(t) + \hat{\mathbf{\Omega}} f(\mathbf{x}(t)) + \epsilon) \text{and} \\
\tilde{\mathbf{c}}(t) &= 1/\tau(\hat{\mathbf{F}}\mathbf{c}(t) + \mathbf{b}),
\end{aligned}$$

implying that an EBN can be trained to implement the dynamics of *any* given RNN obeying the dynamics of Eq. 5.

Let us now restate the dynamics of the spiking network with adapted notation for ease of understanding:

$$\dot{V}(t) = -\lambda V(t) + \mathbf{F}\tilde{\mathbf{c}}(t) - \Omega^{\mathbf{f}}\mathbf{o}(t) + \Omega^{\mathbf{s}}\mathbf{o}(t) + k\mathbf{D}^T \mathbf{e}(t)$$

And let us assume that we have trained an RNN receiving inputs $\mathbf{c}$ to successfully produce a good approximation $\hat{\mathbf{y}} = \hat{\mathbf{D}}\mathbf{x}$ of the target $\mathbf{y}$, so that $\hat{\mathbf{y}} \approx \mathbf{y}$.

We now see that we can train a network of spiking neurons to encode the *dynamics* $\mathbf{x}$ of the RNN, by giving the spiking network input $\tilde{\mathbf{c}}(t) = 1/\tau(\hat{\mathbf{F}}\mathbf{c}(t) + \mathbf{b})$. This makes the *dynamics* of the RNN the new target of the spiking network: $\tilde{\mathbf{x}} = \mathbf{D}\mathbf{r}$, so that $\tilde{\mathbf{x}} \approx \mathbf{x}$. After the recurrent weights $\mathbf{\Omega}^{\mathbf{s}}$ have been learned to implement a network that encodes the rate-network dynamics, classification can be performed by the simple computation

$y(t) = \hat{\mathbf{D}}\mathbf{D}\mathbf{r}(t)$, where $\hat{\mathbf{D}}$ are the rate-network read-out weights, $\mathbf{D}$ are the spiking read-out weights and $\mathbf{r}(t)$ are the filtered spike trains at time t of the spiking network.

Simulation and Learning

To simulate our network, we used a Jax[56] implementation. In all experiments, we used a simple Euler integration method with a time-step of 1 ms.

To ensure that during learning the reconstructed dynamics closely follow the target dynamics, the term $\mathbf{e} = \mathbf{x} - \hat{\mathbf{x}}$ is computed and fed back into the network using the feedforward weights $I_{k\mathbf{D}^T\mathbf{e}} = k\mathbf{F}\mathbf{e}$. The error was then used to compute the update of the slow recurrent weights according to $\dot{\Omega}^{\mathbf{s}} = \eta\mathbf{r}(t)(\mathbf{D}^T\mathbf{e}(t))^T$. In the batched-update version, the filtered spike trains $\mathbf{r}(t)$ and errors $\mathbf{e}(t)$ are then collected in two matrices – $\mathbf{R} \in \mathcal{R}^{N\times T}$ and $\mathbf{E} \in \mathcal{R}^{\hat{N}\times T}$ – which are then used to compute the update $\dot{\Omega}^{\mathbf{s}} = \mathbf{R}(\mathbf{F}\mathbf{E})^T$. In light of the constraint of some neuromorphic chips that reset potentials can not be calibrated on a per-neuron basis, we did not permit updates to the diagonal of $\Omega^{\mathbf{s}}$, which was always set to zero. To furthermore avoid uneven update magnitudes due to the richness of the input (some input signals have only short periods of sound, while others – typically the negative samples – have long ones) we normalised $\dot{\Omega}^{\mathbf{s}}$ by $(\sum_i \sum_j \dot{\Omega}^{\mathbf{s}}_{i,j})/N^2$. Note that this only applies to the batched update case, which we did not use in the experiments.

Finally, the slow recurrent weights are updated according to

$$\Omega^{\mathbf{s}} = \Omega^{\mathbf{s}} + \eta\dot{\Omega}^{\mathbf{s}}$$

where $\eta = 1 \times 10^{-5}$ for the audio task and $\eta = 5 \times 10^{-6}$ for the temporal XOR task.

Temporal XOR task

In this experiment, we trained an RNN with $\hat{N} = 64$ units to implement a classifier for the temporal XOR task. This task consists of a one-dimensional temporal signal comprising two sequentially-presented inputs

of varying sign to be classified according to the logical operator XOR. The result of the XOR is signalled by an output (and target) signal which is zero until the withdrawal of the second input, after which the output should go to ±1 to indicate the result of the XOR. For example, a signal with two positive – or two negative – bumps should be classified as a negative sample and a signal with two bumps that have opposite signs should be classified as a positive example. The temporal nature of the signal makes this task non-trivial, as the network of spiking neurons needs to keep track of the first half of the signal in order to make the correct decision. We then trained a spiking network of $N = 320$ neurons to implement the dynamical system described by the RNN. The parameters of the spiking network are given in Table S2.

TABLE S2
PARAMETERS FOR THE TEMPORAL XOR TASK.

Parameter	*Value*
N_c	1
$\hat{N}$	64
N	320
τ_{mem}	50 ms
τ_{fast}	–
τ_{slow}	70 ms

Network architectures

We investigated the robustness to simulated noise for four different learning paradigms, including the FORCE method, BPTT and reservoir computing. We implemented FORCE, as well as BPTT using Jax[56] as part of Rockpool.[63] To ensure comparability, we chose most of the parameters such as network size and input dimensionality to be the same across different architectures. Table S3 summarises the parameters used for each architecture.

Measurements of parameter mismatch

Using recordings from fabricated mixed-signal neuromorphic chips we measured levels of parameter mismatch (i.e. fixed substrate noise pattern) present in hardware. In particular, for DYNAP-SE,[2] a neuromorphic

TABLE S3

NETWORK ARCHITECTURE PARAMETERS USED DURING NOISE ROBUSTNESS EXPERIMENTS. *: SUBJECT TO MODIFICATION DURING TRAINING.

Parameter	*FORCE*	*BPTT*	*Reservoir*	*ADS Network*
N_c	16	16	16	16
$\hat{N}$	128	–	–	128
N	768	768	768	768
τ_{mem}	10 ms	50 ms*	$\mathcal{U}[1e-4, 0.112]$	50 ms
α	0.00001	–	–	–
τ_{syn}	20 ms	70 ms*	$\mathcal{U}[1e-4, 0.112]$	70 ms/1 ms

processor which emulates LIF neurons with alpha and exponential synaptic response using analog circuits, we measured neuron and synaptic time constants and synaptic weights for individual neuron units, by recording and analysing the voltage traces produced by these circuits. We observed levels of mismatch in the order of 10–20% for individual parameters, with parameter spread being proportional to the mean parameter value (see Fig S1).

Measurements were obtained by capturing traces of membrane voltage of individual silicon neuron circuits of the chip using a program controlled oscilloscope. Even though only the neuron membrane voltage could be observed, manipulations of the neuron and synapse circuit biases allow the measurement of various circuit parameters indirectly.

To measure the neuron time constants, neurons were injected with a square step of constant DC current that leads to a stable membrane potential below the spiking threshold (i.e. membrane leakage is equal to constant input current). After the step input is removed the membrane potential voltage decays to resting state, and the resulting trace was fitted with an exponential decay function to extract the membrane time constant for that neuron.

Refractory periods of neurons were measured by injecting the neurons with sufficient constant DC current to emit spikes. Refractory period was measured as the time between the after-spike membrane potential drop until the membrane potential rose back to the 10% level of the overall trace amplitude.

Synaptic parameters were observed through neuron circuits by setting the neuron time constants to the shortest possible value (i.e. maximizing membrane leakage) so that the membrane voltage directly followed the shape of excitatory and inhibitory synaptic input currents. By stimulating synapses with regular spike trains with low enough rate for the pulses not to interact with each other, the resulting trace amplitude for each pulse amplitude was considered a measure of weight, and the pulse decay time specified the synaptic time constant. Note that the pulse amplitudes should rather be considered as a way of characterizing the relative variability of weights rather their absolute values, as the measurements can only be performed indirectly via the neuron circuit.

Relationship between Alemi[41] and FOLLOW[33] learning rules

The learning rule proposed in ref. 41 closely matches the FOLLOW[33] learning rule for the recurrent updates, when a few assumptions are made.

The learning rule described in ref. 41 is given by

$$\dot{\mathbf{W}}^{\text{slow}} = \Psi(r)(\mathbf{D}^{\mathbf{T}}e)^{T}$$

where $\Psi(r) = \phi(\mathbf{M}r + \theta)$. If we assume $\Psi(r) = r$, which did not have large impact on functionality as demonstrated, we can rewrite the learning rule to

$$\dot{\mathbf{W}}^{\text{slow}} = r(\mathbf{D}^{\mathbf{T}}e)^{T}$$

The authors use the notation that $W_{i,j}$ is the connection from neuron i to j.[41] Since the FOLLOW paper assumes the exact opposite, we rewrite the update rule to

$$\dot{\mathbf{W}}^{\text{slow}} = (\mathbf{D}^{\mathbf{T}}e)r^{T}$$

We now define the error current as

$$I^{\epsilon} = \mathbf{D}^{\mathbf{T}}e = \sum_{\alpha=1}^{\hat{N}} D_{\alpha,i}e_{\alpha}$$

We can now write the update to $\mathbf{W}^{\text{slow}}$ as

$$\dot{W}_{i,j} = \sum_{\alpha=1}^{\hat{N}} D_{\alpha,i}e_{\alpha}(S_j * \kappa) \tag{6}$$

where we replaced r_j with the spike train S_j convolved with the synaptic response kernel κ.

The recurrent update to $W_{i,j}$ in the FOLLOW scheme is given by

$$\dot{W}_{i,j} = (I_i^{\epsilon} * \kappa^{\epsilon})(S_j * \kappa) \tag{7}$$

where $I_i^{\epsilon} = k\sum_{\alpha=1}^{\hat{N}} D_{i,\alpha}^{\text{FOLLOW}} e_{\alpha}$

One can see that the updates 6 and 7 are equivalent under the assumptions $\Psi(r) = r$ and $\kappa^{\epsilon} = \delta$, where δ is the unit impulse kernel.
